# Development of the rehabilitation interventions for people with an acute patellar dislocation in the Physiotherapy Rehabilitation Post Patellar Dislocation (PRePPeD) pilot randomized controlled trial

**DOI:** 10.1302/2633-1462.64.BJO-2024-0174

**Published:** 2025-04-22

**Authors:** Colin P. Forde, Matthew L. Costa, Elizabeth Tutton, Jonathan A. Cook, David J. Keene

**Affiliations:** 1 Oxford Trauma and Emergency Care, Nuffield Department of Orthopaedics, Rheumatology and Musculoskeletal Sciences, University of Oxford, Oxford, UK; 2 Oxford Clinical Trials Research Unit, Centre for Statistics in Medicine, Nuffield Department of Orthopaedics, Rheumatology and Musculoskeletal Sciences, University of Oxford, Oxford, UK; 3 Exeter Medical School, University of Exeter, Exeter, UK

**Keywords:** Patella, Patellofemoral dislocation, Patellar dislocation, Patellar instability, Rehabilitation, Physical therapy, Exercise, acute patellar dislocations, patellar dislocations, physiotherapy, randomized controlled trial, Physical therapists, supervised rehabilitation, exercise programmes, knee, muscle strength

## Abstract

**Aims:**

To develop the rehabilitation interventions for people with an acute patellar dislocation in the Physiotherapy Rehabilitation Post Patellar Dislocation (PRePPeD) pilot randomized controlled trial (RCT), and to describe how these interventions are delivered.

**Methods:**

We developed the interventions drawing on a range of established intervention development approaches and frameworks. We selected intervention components after reviewing the existing evidence, clinical guidelines, UK NHS practice, and relevant scientific theory. We then created early versions of the interventions, and discussed these with clinical experts and patient and public partners. We finalized the interventions considering their feedback, findings from our preliminary study, and what would be acceptable and deliverable in the UK NHS.

**Results:**

Upon randomization, all participants receive a workbook containing advice and initial exercises to implement before their first physiotherapy session. Self-managed rehabilitation then involves a single one-to-one session with a physiotherapist who provides advice, introduces a structured home exercise programme, and uses strategies to support exercise adherence. Participants then continue their recovery independently. Supervised rehabilitation involves four to six one-to-one physiotherapy sessions over a maximum of six months. Physiotherapists also provide advice, prescribe home exercise, and use exercise adherence strategies. Routine follow-up sessions enable physiotherapists to reassess participants and tailor the advice and exercises accordingly.

**Conclusion:**

The interventions were developed and are currently being assessed in the PRePPeD pilot RCT. This will determine whether a full-scale RCT comparing these interventions is feasible. Results are anticipated in Summer 2025.

Cite this article: *Bone Jt Open* 2025;6(4):469–479.

## Introduction

The reported incidence of first-time patellar dislocations is 2.58 to 42.0 per 100,000 person-years at risk, highest in the second decade of life, and equal for males and females.^1,2^ After a first-time dislocation, about one in four people redislocate their patella within ten years.^2^ In the UK, initial management of isolated acute patellar dislocations is usually non-surgical.^3^ Rehabilitation is then recommended to help patients recover.^3^ Rehabilitation aims to restore function and reduce recurrent instability episodes by improving modifiable impairments, for example in knee joint movement, lower limb muscle strength, and dynamic control.^4^ Despite being routinely provided, there is no high-quality evidence to inform rehabilitation practice^5^ and physiotherapists’ treatment,^6^ and patient outcomes vary.^5,6^ High-quality rehabilitation trials are needed to inform clinical practice. However, before evaluating new interventions, complex intervention development guidance recommends that new interventions should be rigorously developed to improve their chances of being effective.^7^ Publishing how interventions are developed and delivered is also recommended to enable interpretation and implementation of the results of any future evaluation.^7^

Previously, our preliminary study showed that a prototype intervention of up to six physiotherapy sessions of tailored advice and exercise appeared acceptable to adults after acute patellar dislocation and was deliverable in the UK NHS.^8^ However, high-quality trials for other musculoskeletal conditions have shown that multiple physiotherapy sessions of tailored advice and exercise are not necessarily more effective than one session that enabled self-management.^9,10^ The Physiotherapy Rehabilitation Post Patellar Dislocation (PRePPeD) study is assessing the feasibility of a full-scale randomized controlled trial (RCT) comparing these two rehabilitation approaches. PRePPeD is a parallel-group, external pilot RCT and embedded qualitative study comparing supervised (four to six physiotherapy sessions) versus self-managed rehabilitation (one physiotherapy session) for people aged ≥ 14 years with an acute first-time or recurrent patellar dislocation. Further details are in the published protocol.^11^ Here, we describe the development and delivery of the interventions for the PRePPeD study.

## Methods

**Fig. 1:**
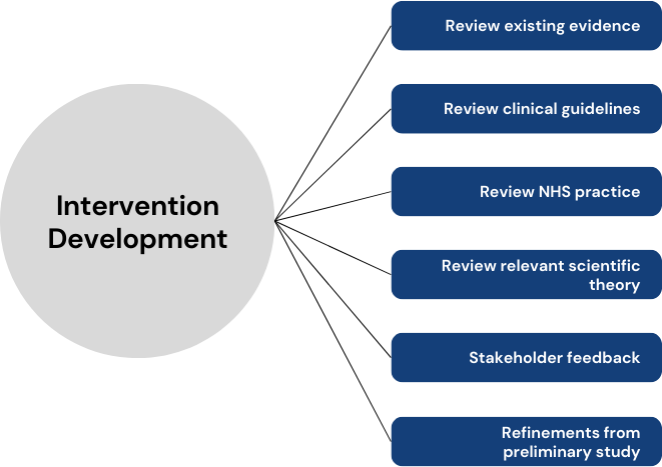
Overview of the intervention development process.

We developed the PRePPeD interventions drawing on a range of established intervention development approaches and frameworks.^12^ We selected intervention components after reviewing the existing evidence, clinical guidelines, NHS practice, and relevant scientific theory. We then created early versions of the interventions and discussed these with clinical experts and patient and public partners. We created the final interventions considering their feedback, our preliminary study’s findings, what would be acceptable to patients and clinicians, and what would be deliverable in the NHS. Figure 1 presents an overview of the intervention development process. Although presented sequentially, this was an iterative process.

### Intervention development

#### Phase 1a: existing evidence

A 2018 systematic review identified only one RCT that evaluated rehabilitation after the initial immobilization period for people with a lateral patellar dislocation.^5^ This study found a statistically significant but clinically questionable difference in function and activity levels between two different quadriceps-strengthening exercise programmes, but there were limitations in sample size (n = 50) and retention rates (48%).^13^ To identify new studies reporting non-surgical treatment, we updated this review’s MEDLINE and Embase search strategy in April 2022. We retrieved seven relevant studies (see Supplementary Material for the search strategy and synthesis of non-surgical interventions)^14-20^ but only one RCT, which is ongoing.^14^ This RCT is comparing actual with sham blood flow restriction training alongside standard physiotherapy, which involves 24 physiotherapy sessions. This far exceeds the volume of physiotherapy normally commissioned in the NHS, so the results are unlikely to influence NHS practice.

The available literature shows that there is insufficient evidence to support any aspect of acute patellar dislocation rehabilitation after initial immobilization. Published interventions vary and are often poorly reported. Where details are provided, interventions mainly involve advice and exercises to improve knee joint movement and lower limb muscle strength. However, none (excluding our preliminary study) prescribed resistance exercises using evidence-based prescription guidelines,^21^ retrained movement strategies to reduce reinjury risk, or used behaviour-change strategies to support exercise adherence.

#### Phase 1b: clinical guidelines

At the time of intervention development, three English-language guidelines referred to rehabilitation for people with patellar dislocations/instability. The British Orthopaedic Association recommends that rehabilitation after patellar stabilization surgery should include education/advice, restore range of motion, address lower limb control during functional movements, and involve prescription of a functional tailored exercise programme.^22^ The accompanying paper explained that detailed guidance is lacking due to variable practice and insufficient high-quality evidence.^23^ Guidance from Italy recommends that initial non-surgical treatment for people with acute first-time patellar dislocations should include pharmacological pain and inflammation management, temporary immobilization, and crutch use.^24^ Exercise is considered central to recovery, but the lack of evidence to guide exercise prescription is highlighted.^24^ Finally, the American Orthopaedic Society for Sports Medicine and the Patellofemoral Foundation recommends that the contribution of movement patterns to patellar instability should be investigated.^25^ These guidelines demonstrate that exercise is deemed integral to rehabilitation, but specific guidance about exercise type, dose, and mode of delivery is lacking.

#### Phase 1c: NHS practice

##### Physiotherapists’ practice

A survey of NHS physiotherapists identified that treatment for people after first-time patellar dislocation varies, but predominantly involves advice (about rest/activity modification and reassurance) and prescribed exercise (mainly knee joint movement, proprioception, and lower limb muscle strengthening exercises).^6^ Treatment lasts from three to six weeks (24% of physiotherapists) to at least four months (12%), and is delivered either one-to-one (51%) or both one-to-one and in a group (48%).^6^ These findings demonstrate that advice and exercise are the core aspects of NHS physiotherapists’ treatment, but there is inconsistency in prescribed exercises, treatment durations, and mode of delivery.

##### Publicly available information

To enhance our understanding of non-surgical treatment in the NHS, we searched Google (USA; first ten result pages) in April 2022 for publicly available NHS information about patellar dislocations. We retrieved 16 relevant resources from 14 NHS organizations, one private NHS provider, and the NHS website. Resources focused on the acute injury phase and mainly contained advice and exercise, underlining that these are the key components of non-surgical treatment in the NHS. The types of advice and exercises in retrieved resources informed the content of acute injury advice and initial exercises in intervention materials. The Supplementary Material contains the search strategy and synthesis of advice and exercises in retrieved resources.

### Phase 1d: scientific theory

#### Acute injury management

Local pain, swelling, and inflammation are normal parts of the acute patellar dislocation injury response, but if prolonged or excessive they can affect recovery. For example, knee pain and swelling reduce quadriceps muscle strength and activation.^26^ This could impair lower limb muscle strength restoration with implications for recovery. Excessive swelling can also contribute to secondary ischaemic injury,^27^ and reduce joint flexibility, impeding function. Therefore, we included pain and swelling management advice that was informed by acute soft-tissue injury management guidance.^28^

#### Exercise

Most patellar dislocations are non-contact injuries thought to occur when the foot is planted,^29^ the knee is flexing from an extended position, and a valgus/rotational force is applied to the knee.^24,30^ This increases the lateral orientation of the resultant eccentric quadriceps contraction, which if sufficient to overcome the restraints to lateral patellar translation, dislocates the patella.^30^ Normally, the medial patellofemoral ligament is torn.^31^ This is the main soft-tissue restraint to lateral patellar translation in early knee flexion,^32^ so patients are more vulnerable to recurrent instability in this position, particularly during high quadriceps-demand activities (e.g. single leg landing). The interventions therefore include exercises that aim to retrain movement strategies during functional movements to reduce reinjury risk. This mainly involves modifying trunk and lower limb alignment to reduce the magnitude and lateral orientation of the quadriceps force.

Improving muscle strength, particularly of the hip and thigh muscles, is also a key focus because of their role in controlling motion and loads at the hip and knee. Restoring thigh muscle strength could also help maintain long-term knee health. Stronger quadriceps muscles were associated with less patellofemoral cartilage loss in people with knee osteoarthritis.^33^ This is relevant for this population of patients, who have an increased risk of developing patellofemoral osteoarthritis.^34^

#### Strategies to support exercise adherence

Participant adherence to prescribed exercise is important for exercise-based interventions to be effective. Yet, patient-reported adherence to physiotherapy prescribed exercise is estimated to be 67%.^35^ Consequently, the interventions use behaviour-change strategies from our preliminary study, which were associated with high participant-reported exercise adherence.^8^ These strategies are informed by systematic review evidence^35,36^ and the NHS Health Trainer Handbook,^37^ which are informed by behaviour-change science.

### Phase 2: stakeholder feedback

Following phase 1 and drawing on our clinical experience, we created an initial outline of the self-managed and supervised rehabilitation interventions (Supplementary Material), and discussed these with expert physiotherapists and patient and public involvement (PPI) partners. Given the limited evidence to inform rehabilitation practice, their feedback was important to optimize the interventions and increase the chances that they would be acceptable and implementable in the NHS.

#### Expert physiotherapists

Six UK physiotherapists with expertise in patellar dislocation rehabilitation (see Acknowledgements) gave feedback on the proposed interventions during an online group (four physiotherapists) or a one-to-one meeting (two physiotherapists). Physiotherapists made several recommendations, which we implemented. For instance, initial physiotherapy sessions are face-to-face to allow physiotherapists to conduct a physical assessment and build rapport with participants, whereas follow-up sessions can be face-to-face or by video. Later, three physiotherapists from participating sites reviewed the supervised and self-managed rehabilitation workbooks, which resulted in minor amendments to the content.

#### PPI partners

The National Institute for Health and Care Research (NIHR) GenerationR Liverpool Young Persons Advisory Group^38^ and two adults with a previous patellar dislocation provided a patient’s perspective on the proposed interventions. PPI partners agreed with physiotherapists that initial physiotherapy sessions should be face-to-face and follow-up sessions either face-to-face or by video. They also felt that participants allocated to the ‘self-managed rehabilitation’ intervention should be able to initiate one follow-up session if they struggle with their exercises, believing this would increase intervention acceptability. There was also consistent feedback that creating exercise videos could improve exercise adherence. All these suggestions were implemented.

### Phase 3: refinements from our preliminary study

For the pilot RCT, we adapted the intervention by increasing the maximum treatment duration of the supervised rehabilitation intervention from three to six months based on participants’ reported preferences in the preliminary study.^8^ Participants allocated to supervised rehabilitation also receive four to six physiotherapy sessions to ensure that there is a clear difference in the number of physiotherapy sessions between interventions, which was an issue in a similar rehabilitation trial.^39^ We have also used an alternative method for setting resistance exercise intensity due to poor physiotherapist compliance with a modified Borg scale in the preliminary study. Additional refinements included removing exercise options that were infrequently prescribed and streamlining the exercise adherence strategies for time efficiency (e.g. removing a behavioural contract).

### The interventions

The interventions are reported following the Template for Intervention Description and Replication Guidelines,^40^ and the Consensus on Exercise Reporting Template.^41^

#### Intervention delivery

Upon randomization, which typically occurs in outpatient fracture/knee clinics, a clinician/researcher gives the participant a paper self-managed or supervised rehabilitation workbook according to their allocation. The first section of both workbooks is the same, and contains advice (Table I) and initial exercises (Table II) that participants are advised to implement immediately. The participant is then referred to physiotherapy and told to bring their workbook to the sessions. Providing intervention materials before physiotherapy aims to accelerate recovery by minimizing pain, swelling, and the consequences of prolonged inactivity.

**Table I.** Overview of advice in section one of the workbooks.

Section	Contents
Introduction	Explains the contents and purpose of the workbook
About your kneecap	Explains basic patellofemoral anatomy using text and pictures
What is a kneecap dislocation?	Describes what a kneecap dislocation is and common injury mechanisms
What can I expect after a kneecap dislocation?	Common symptoms after injury
What can I do to help my knee get better?	Why managing pain is important and how to do this (including medication and using cold) Why managing swelling is important and how to do this (including elevating the injured leg) Benefits of restoring normal activities gradually and the negative consequences of fear-avoidance behaviours Flare-up management
Walking and stairs	Explains the benefits of gradually restoring normal gait, weightbearing, and stair climbing, and how to do this. Pictures/videos and instructions on walking and stair climbing with crutches provided
Knee splints	Guidance on when any knee splints provided can normally be removed
Exercise guide	How exercise helps recovery Guidance on permissible symptoms during and after exercise The stretching, balance, and strengthening exercises to start before the first physiotherapy session with pictures/videos and instructions on technique and progressions
Physiotherapy	How the physiotherapy appointment will be arranged, what physiotherapy will involve, and its benefits
What to do if you have any problems or haven’t receive a physiotherapy appointment	Expected timelines for recovery. Advice on who to contact if participants are experiencing problems or have not received their physiotherapy appointment

**Table II.** Overview of initial exercises in section one of the workbooks.

Category	Level	Description	Prescription parameters	Progression
**Stretching**				
Knee bending	1	AROM knee flexion in sitting	3 × 10 reps	Bend knee further using unaffected leg
	2	AAROM knee flexion in long sitting using hands	3 × ≥ 30 s	Bend knee further
Knee straightening	1	Static quadriceps muscle contractions	3 × 10 reps	Push knee down harder
	2	Static quadriceps muscle contractions with foot elevated (e.g. on rolled up towel)	3 × 10 reps	Push knee down harder
**Balance**	1	Weight shifting with upper limb support	3 × 10 reps	Increase weight through affected leg, increase time up to 10 s
	2	Single leg stand with contralateral upper limb support	3 × 10 reps	Lean on support less, increase time per rep up to 10 s
	3	Single leg stand without upper limb support	3 × 10 reps	Increase time per rep up to 10 s
**Strengthening**	1	Squat to chair	3 × 10 reps, ≥ 1 min between sets	Increase weight through affected leg, use lower chair
	2	Squat to chair split stance (unaffected leg in front)	3 × 10 reps, ≥ 1 min between sets	Reduce weight on unaffected leg

AAROM, active assisted range of motion; AROM, active range of motion; PROM, passive range of motion; rep, repetition; s, seconds.

The second section of the workbook differs between treatment groups, and contains the additional advice, exercises, and exercise adherence materials that physiotherapists use to deliver the interventions at treatment sessions. Initial physiotherapy sessions are as soon as possible after randomization, ideally within three weeks. This aims to ensure that physiotherapy starts in the acute phase of injury, normally considered within six weeks (participants are recruited ≤ 21 days of injury).

Participants whose contact details include an email address are automatically emailed access to an online version of section one of the workbook. The only difference from the paper workbook is the availability of videos on using crutches and performing exercises. We did not create an online version of section two of the workbook because not all NHS departments can deliver interventions using exclusively online materials. However, all intervention exercises are accessible in video format via a QR code or URL in paper workbooks. We created online materials because we anticipated recruiting a young participant population, and ≥ 96% of UK 16 to 34-year-olds use a smartphone for private use.^42^

#### Intervention providers

Fully qualified NHS physiotherapists provide the interventions at participating sites’ physiotherapy departments (for face-to-face appointments). Physiotherapists do not have to possess certain experience or expertise, but this is recorded for study reporting.

Before providing the interventions, physiotherapists receive two-hour face-to-face or online training covering the study background, an overview of the interventions, intervention delivery, and study documentation. Physiotherapists can provide both interventions, but to limit treatment contamination the interventions are highly structured and intervention delivery is closely monitored (see ‘quality assurance’ section). A previous trial in our group implemented this approach and found no evidence of contamination.^39^ To promote intervention fidelity, physiotherapists receive a manual detailing intervention delivery and are encouraged to contact the trial team with any queries.

#### Supervised rehabilitation

**Fig. 2:**
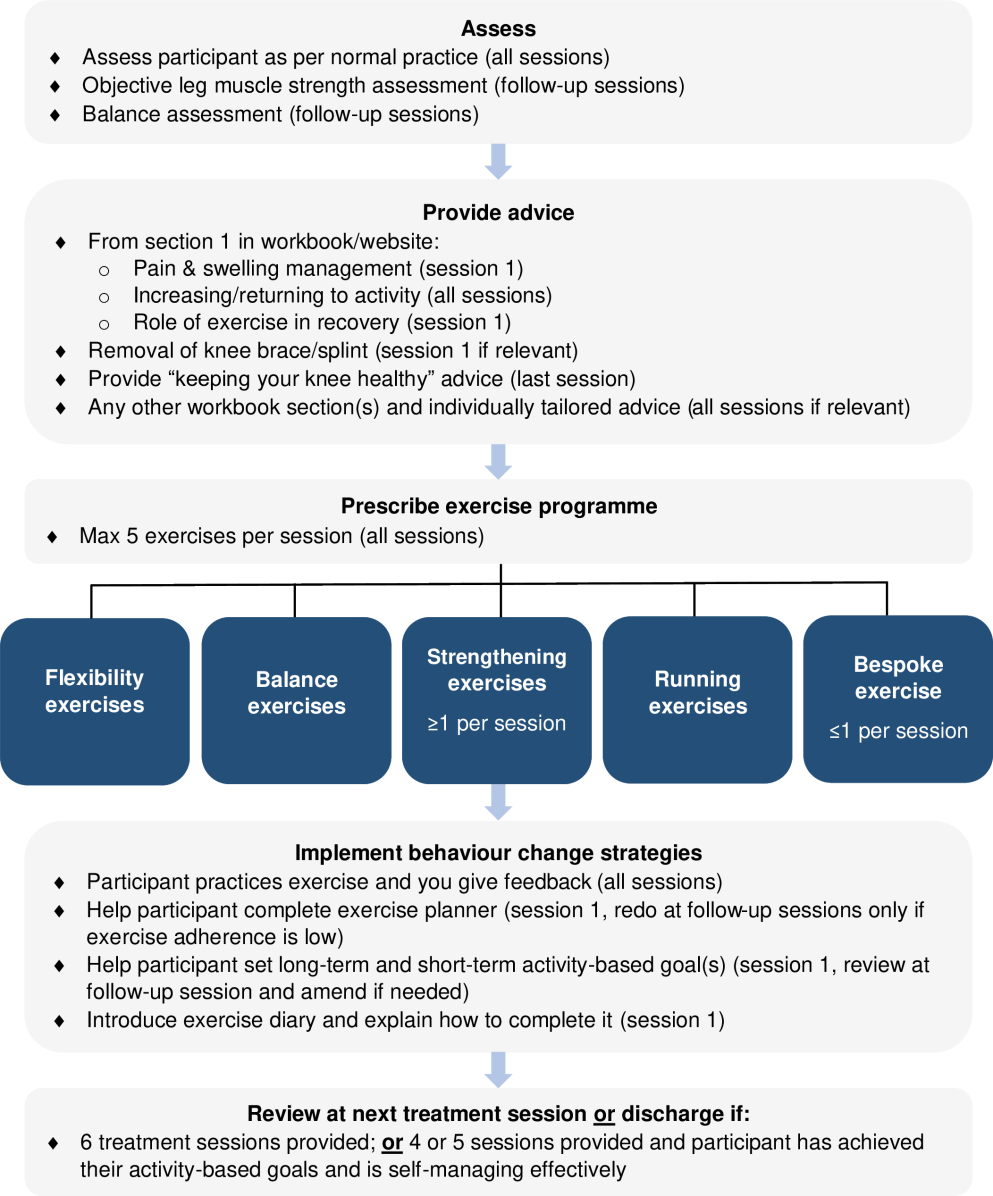
Overview of the supervised rehabilitation intervention delivery process.

An overview of the supervised rehabilitation intervention delivery process is presented in Figure 2.

Supervised rehabilitation involves four to six physiotherapy sessions over a maximum of six months at a frequency decided between physiotherapists and participants. Initial sessions are face-to-face and last ≤ 60 minutes, and follow-up sessions are ≤ 30 minutes and are face-to-face or by video depending on the physiotherapist’s and participant’s preferences. Participants are discharged after six sessions, or after four or five sessions if they achieve their goals and are self-managing effectively.

The key differences in this intervention are that physiotherapists can reassess participants at follow-up sessions and have greater autonomy over exercise prescription. This aims to encourage physiotherapists to provide advice and prescribe exercises that are informed by their assessment findings and clinical expertise, and tailored to participants’ physical characteristics, stage of recovery, and activity goals.

##### Assessment

Physiotherapists assess participants following their normal practice. At follow-up sessions, physiotherapists also assess participants’ objective leg muscle strength and balance once this is judged safe (the term ‘balance’ is used for ease of reference, but this could refer to an assessment of static balance or functional movement strategies). This aims to monitor recovery of these key physical performance variables and to guide exercise prescription. We require sites to use an objective muscle strength assessment method because manual muscle testing underestimates between-limb differences.^43^ Any balance and objective leg strength assessment method is permitted but these are recorded to inform future intervention iterations. We provide sites without objective muscle strength testing equipment with a hand-held dynamometer (Lafayette model 001165; Lafayette, USA) and guidance on how to use it. Intervention training encourages physiotherapists to select a balance assessment method considering participants’ stage of recovery, physical capabilities, and the demands of activities that they plan to resume.

##### Advice

At session one, physiotherapists re-emphasize the advice in the workbook about pain and swelling management, resuming normal activities, the role of exercise in recovery, and removing the knee splint/brace (if applicable). At follow-up sessions, the importance of restoring pre-injury activities is emphasized. On the day of discharge, physiotherapists introduce advice about ‘keeping your knee healthy’ from section two of the workbook, which is based on clinical guidelines for non-pharmacological management of osteoarthritis.^44^ We included this advice because this patient population has a high risk of recurrent dislocation, an increased risk of developing patellofemoral osteoarthritis, and the physiological adaptations to exercise are reversible when exercise is stopped.^45^ Throughout, physiotherapists can highlight any other advice from the workbook or provide any other advice deemed relevant.

##### Exercise

Physiotherapists prescribe exercises from a menu in the participant’s workbook. Exercises are divided into four categories: stretching, balance, leg strengthening, and running (Supplementary Material). Physiotherapists can prescribe a maximum of five exercises per session, because more than five has been associated with reduced exercise adherence.^46^

In keeping with the focus on improving lower limb muscle strength, physiotherapists must prescribe at least one leg-strengthening exercise per session following exercise prescription guidelines (one to three sets of eight to 12 repetitions ≥ three times a week with ≥ two minutes rest between sets).^21^ To ensure strengthening exercises are sufficiently intense to stimulate continued improvements (i.e. the progressive overload principle),^21^ participants are advised to increase the load if they can perform three sets of 12 repetitions and do two extra repetitions after the last set for two consecutive sessions.^21^ For all other exercise categories, physiotherapists prescribe exercise parameters.

Physiotherapists can prescribe one exercise per session that is not on the PRePPeD exercise menu if deemed important to help participants achieve their goals. This aims to cater for variability in participant goals and to keep the exercise menu concise enough to be user-friendly. Prescribed bespoke exercises are recorded for study reporting and to inform future intervention iterations.

All exercise sheets contain written instructions, pictures, and optional sections where physiotherapists can write ‘make harder by’ and ‘other tips/advice’ instructions. The sheets of prescribed exercises are placed in a plastic pocket in the participant’s workbook labelled ‘current exercises’, and physiotherapists change these at each session as required.

##### Strategies to support exercise adherence

At session one, participants write in an exercise planner where and when they will do their exercises along with a contingency plan, set short- and long-term SMART (specific, measurable, achievable, relevant, timely) goals, and physiotherapists introduce an exercise diary where participants record exercise performance. At follow-up sessions, physiotherapists review participants’ exercise diaries and goals. If adherence to prescribed exercise is low, participants complete the exercise planner again. Similarly, participants amend their goals if the physiotherapist’s review indicates that this is necessary. Throughout, participants practise prescribed exercises and receive feedback on technique, and physiotherapists provide any support with exercise adherence strategies that participants need.

### Self-managed rehabilitation

**Fig. 3:**
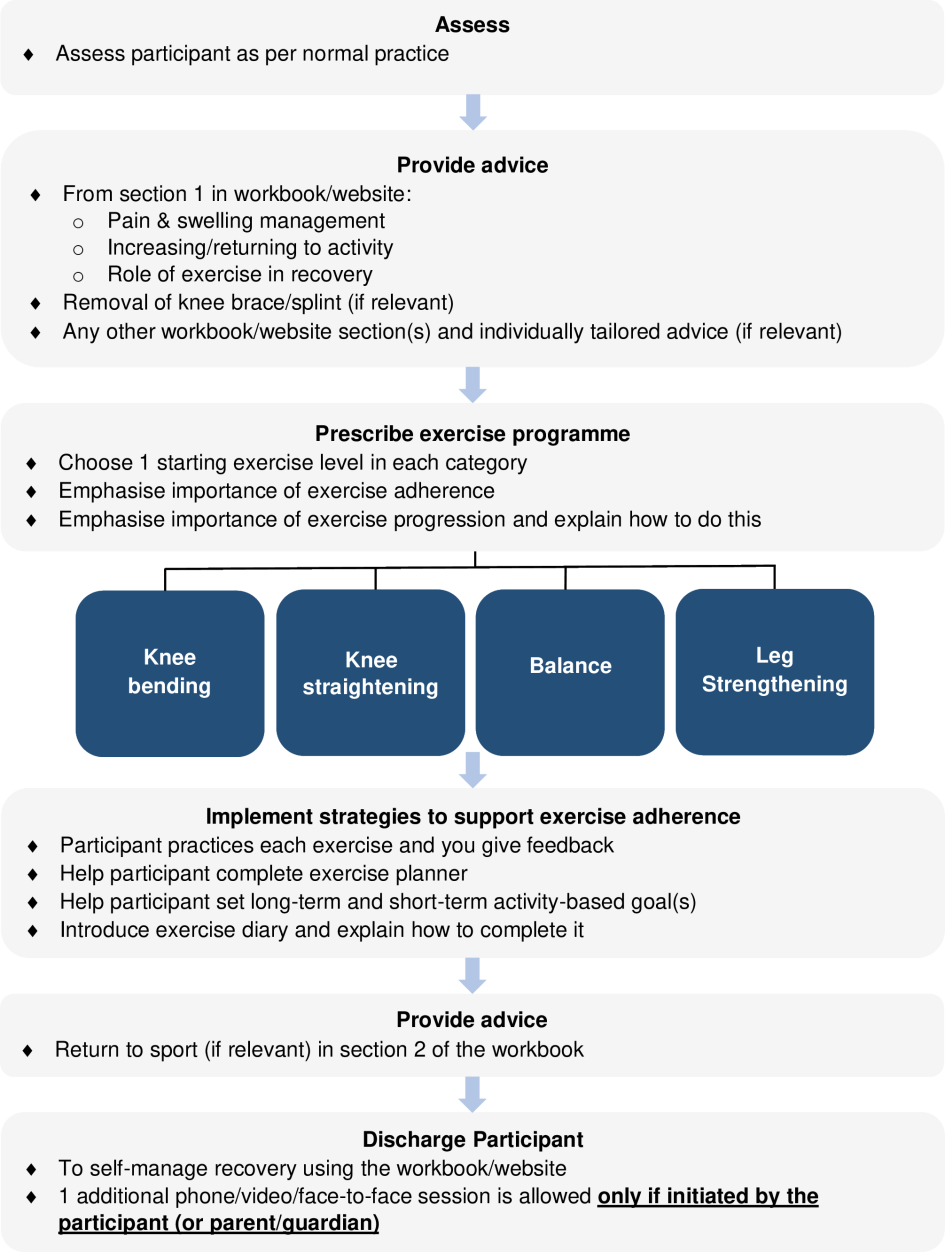
Overview of the self-managed rehabilitation intervention delivery process.

An overview of the self-managed rehabilitation intervention delivery process is presented in Figure 3.

Participants receive a single, face-to-face, one-to-one physiotherapy session lasting ≤ 60 minutes, then continue their recovery independently. Participants who struggle with exercise progression can contact their physiotherapist for advice, or to schedule one additional physiotherapy session by phone, video, or face-to-face.

#### Assessment

Physiotherapists assess participants as per their normal practice.

#### Advice

Physiotherapists re-emphasize the same advice in the workbook as described for session one in supervised rehabilitation. In addition, if participants plan to return to sport, physiotherapists introduce advice in section two of the workbook about how to do this safely. We included this guidance because this is mainly a young patient population, and two-thirds of patellar dislocations occur during sports.^1^

#### Exercise prescription

The self-guided exercise programme is composed of knee bending, knee straightening, balance, and strengthening exercises (Supplementary Material). Each category contains exercises of progressively challenging levels of difficulty (level one is the easiest). Using their assessment findings and clinical judgement, physiotherapists prescribe a starting exercise level in each category (i.e. a total of four exercises). Physiotherapists then explain the workbook advice on how to progress through the exercise levels until participants reach their desired or pre-injury level, and the importance of exercise progression for recovery. We recommend that participants perform exercises at least three times per week for three months in keeping with the recommended frequency^21^ and duration required for neuromuscular adaptation^47^ to resistance training. Participants are advised to perform exercises more often and for longer if helpful, and that performing strengthening exercises one to two times per week is necessary to maintain long-term muscle strength.^21^

#### Strategies to support exercise adherence

Like supervised rehabilitation, participants practise prescribed exercises and receive feedback, plan where and when to perform exercise, set goals, and record exercise performance in a diary. The difference is that there are no follow-up sessions where the physiotherapist reviews adherence and goals, and provides support to amend these if necessary.

### Other treatments

Physiotherapists can provide other treatments (e.g. taping) during intervention sessions as long as the core intervention components described above are provided. Additional treatments are recorded in physiotherapist-completed treatment logs for study reporting and to inform future intervention iterations. Participants’ use of additional physiotherapy sessions, including out-of-trial NHS and private physiotherapy, are recorded and monitored. Sites notify general practitioners (GPs) of their patient’s participation because GPs can refer to physiotherapy.

### Quality assurance

At least one treatment session is observed or audio recorded per site. We also review physiotherapist-completed treatment logs that record the session number, mode of delivery, duration, and content. Where required, we provide feedback to sites and individual physiotherapists, provide additional intervention training, or conduct additional observation/audio recording of treatment sessions. Full details about the intervention quality assurance activities are in the study protocol.^11^

## Discussion

To our knowledge, this is the first paper to describe the development of rehabilitation interventions for people with an acute patellar dislocation. We developed the interventions rigorously, drawing on a range of established intervention development approaches, sources of evidence, and stakeholder feedback. This transparent reporting follows Medical Research Council/NIHR-funded guidance that recommends publishing how complex healthcare interventions are developed and are delivered.^48^ This will enable others to understand the rationale for the interventions, and enable interpretation and implementation of the results of a future full-scale evaluation if this is undertaken.

The interventions are currently being evaluated in the PRePPeD pilot trial and embedded qualitative study. Results are anticipated in Summer 2025. If a full-scale trial is feasible and undertaken, further intervention refinement may be needed contingent on our pilot trial and qualitative study findings, new evidence, and wider stakeholder input. This future full-scale trial’s results would provide high-quality evidence to guide rehabilitation provision for people with an acute patellar dislocation treated in the UK NHS.

**Take home message**

- We have described how the interventions for the PRePPeD pilot trial were developed and are delivered. This will enable readers to understand the evidence, scientific theory, and rationale underpinning the main interventions components.

- It will also facilitate the implementation of the results of a future full-scale trial comparing these interventions, if this is undertaken.

## Data Availability

The data that support the findings for this study are available to other researchers from the corresponding author upon reasonable request
